# Adequacy of Semitendinosus Tendon Alone for Anterior Cruciate Ligament Reconstruction Graft and Prediction of Hamstring Graft Size by Evaluating Simple Anthropometric Parameters

**DOI:** 10.1155/2012/424158

**Published:** 2012-07-29

**Authors:** Papastergiou G. Stergios, Konstantinidis A. Georgios, Natsis Konstantinos, Papathanasiou Efthymia, Koukoulias Nikolaos, Papadopoulos G. Alexandros

**Affiliations:** ^1^Orthopaedic Department of “Saint Paul” General Hospital of Thessaloniki Greece, Ethnikis Antistaseos 161, 55134 Thessaloniki, Greece; ^2^Department of Anatomy, Medical School, Aristotle University of Thessaloniki, University Campus, P.O. Box 300, 54624 Thessaloniki, Greece

## Abstract

*Introduction*. Preoperative identification of patients with inadequate hamstring grafts for anterior cruciate ligament reconstruction is still a subject of interest. *Purpose*. The purpose of this study was to determine whether the semitendinosus tendon length is adequate for four-strand graft harvested by common technique (without bone plug) and whether there is correlation of gracilis and semitendinosus tendon grafts length and diameter of quadrupled graft with anthropometric parameters. *Materials and Methods*. In this retrospective study, 61 patients (45 males, 16 females) undergoing ACL reconstruction using four-strand hamstring autograft tendons were included. *Results*. The length of semitendinosus tendon, harvested by the common technique, was in 21% of our cases inadequate in order to be used alone as a four-strand graft especially in females (43%). There was moderate correlation between semitendinosus and gracilis graft diameter and patient's height and weight and fair correlation to BMI. We found no statistically important predictor for graft diameter in female patients. *Conclusions*. The length of semitendinosus tendon, harvested by common technique, is usually inadequate to be used alone as a four-strand graft especially in females. The most reliable predictor seems to be patient's height in males. In female patients, there is no statistically important predictor.

## 1. Introduction

The anterior cruciate ligament (ACL) is the most commonly reconstructed ligament of the knee [1]. An injury to the ACL can result in significant functional impairment [2]. Strength and stiffness of the graft are important components in order to decide the kind of graft and the technique of tendon replacement.

It is widely accepted that four-strand hamstring autograft represents a successful option for ACL reconstruction [3–7]. A possible complication when using both the semitendinosus (ST) and gracilis (G) tendon graft is that of hamstring strength deficit in deep flexion and internal rotation [8–10]. Gobbi and Francisco suggest to use only ST tendon in a four-strand graft with bone plug in order to reduce donor's site morbidity and to increase graft's diameter [11], while later on in another study Gobbi again suggests a double bundle using only semitendinosus tendon for better functional rehabilitation of the knee [12]. In this type of operations, it would be essential for the surgeon to be able to predict preoperatively graft length in order to choose the ideal graft type and to avoid scar formation, pain, operating time and infection risk. Prediction of graft length could also be useful in cases of revision ACL reconstruction where usually a larger diameter graft is needed [13] or in cases of active and/or highly demanding patients or professional athletes where larger diameter grafts would be ideal. 

Scott and Insall report that the length of normal ACL is 38 mm (25–41 mm) and the width is 10 mm (7–12 mm), on average [14]. In order to assure the optimal 7 cm quadrupled graft construct for ACL reconstruction (2 cm in the femoral tunnel, 3 cm intra articular, and 2 cm in the tibial tunnel), it is essential to obtain a minimum tendon length of 28 cm (ranged from 28 to 30 cm) with a minimum thickness of 7 mm [11, 15–17].

The purpose of this retrospective study was to determine whether alone the ST tendon length is adequate for four-strand graft harvested by common technique and whether there is correlation of G and ST grafts length and diameter with anthropometric parameters. 

## 2. Materials and Methods

Sixty-one consecutive patients (45 males—16 females) undergoing ACL reconstruction using four-strand hamstring autograft tendons were included in this retrospective study. Age, gender, height, weight, and Body Mass Index (BMI) for each patient were recorded preoperatively.

**Figure 1:**
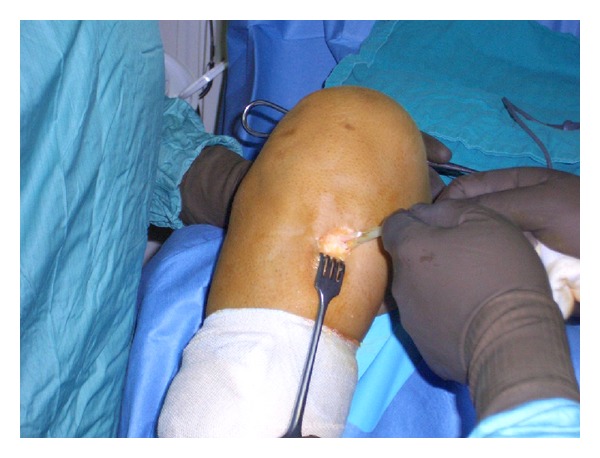
Hamstring tendons harvesting in the pes anserinous attachment area on the proximal tibia.

**Figure 2:**
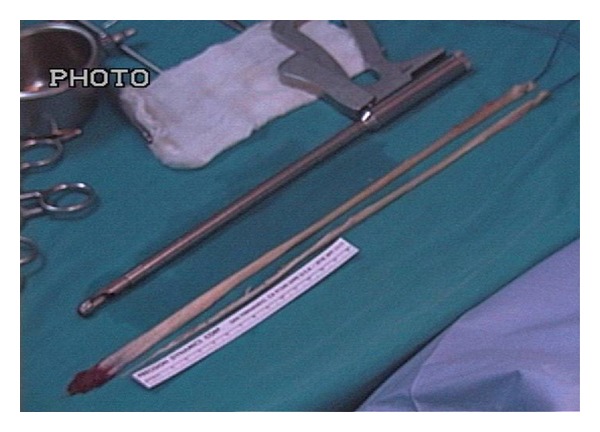
Excised ST and G tendon with a close tendon stripper. Photo is taken with dyonics arthroscopic video camera.

**Figure 3:**
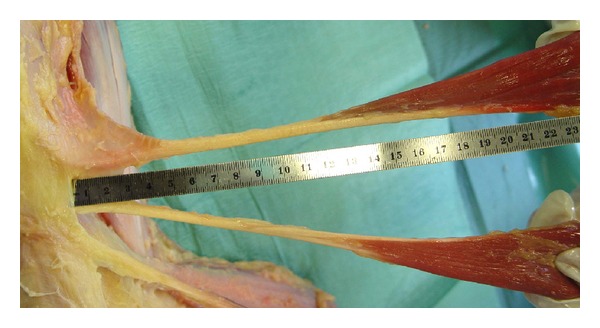
Anatomical dissection of ST and G tendons attachment, that shows their maximum lengths in a cadaveric specimen.

**Figure 4:**
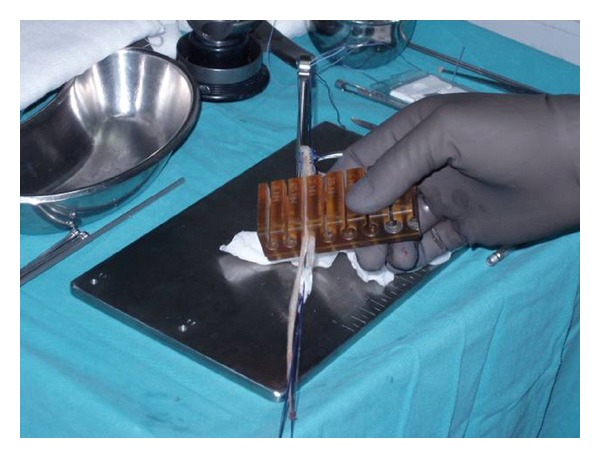
Intraoperative diameter measurement of a quadrupled ST and G tendon graft using sizing cylinders with incremental size change of 0.5 mm.

Surgical operation was performed by the same surgeon in all cases, and hamstring tendon autografts (ST-G) were harvested by the same way. An oblique incision was performed on the skin over the pes anserinus attachment area on the proximal tibia. Subcutaneous fat was incised and attentive blood hemostasis was performed. The sartorius fascia was incised parallel to the direction of G tendon. On the next step, G tendon was dissected (Figure 1). The tendon was removed from its proximal attachment with a close tendon stripper (Figure 2). The detachment of the tendon on its tibial end was done close to the bone in order to preserve its maximum length (Figure 3). The same procedure was followed for ST tendon but before removal of its proximal attachment, the tendon band towards gastrocnemius muscle was dissected with a scissor under direct vision. After both hamstring tendons harvest each one of them became double strand to create a four-strand graft with both tendons. Each end of the tendon grafts was stitched with a no. 2 nonabsorbable polyester suture. After blunt removal of attached muscle and fat but before any further postharvested alteration of trimming of the graft, intraoperative measurements of both tendons were done, such as length of each and diameter of the quadrupled graft using sizing cylinders with incremental size change of 0.5 mm. The graft diameter was considered to be the dimension of the smallest cylinder that could pass through (Figure 4). In case that the graft diameter was not exactly fitted in a specific 0.5 mm increment, the size of the graft was recorded according to the drill size of our tunnels. Pretensioning of the graft on the surgical table was not performed. Finally single-bundle ACL reconstruction was performed.

Adequate length of ST tendon graft as only four-strand graft for single-bundle ACL reconstruction was considered to be 28 cm. Adequate diameter of four-strand hamstring autograft (ST-G) was considered to be more than 7 mm. 

In statistical analysis, independent samples *t*-test was used to identify differences between the mean values of continuous variables according to gender. Chi-square statistics (*χ* ^2^) was done to investigate any possible association of the categorical variables with the diameter of the graft. Bivariate correlation coefficients (Pearson *r*) and multiple linear regressions were calculated to evaluate any possible association between clinical data and intraoperatively measured hamstring graft lengths and diameters. Higher correlation coefficients indicate stronger relationships between variables. Statistical package SPSS version 18.0; SPSS, Chicago, III for Windows was used for analyses. A *P* value of 0.01 was taken as the level of significance. 

## 3. Results

Anthropometric measurements including the average age, weight, height and BMI, and gender of patients participating in this study are shown in Table 1. Graft characteristics are described according to gender in Table 2. Frequency of adequate ST tendon graft length according to gender is presented in Table 3. Frequency of adequate four-strand hamstring (ST-G) autograft diameter according to gender is summarized in Table 4.

Female patients were lighter and shorter with lower BMIs, and had shorter length and smaller diameter hamstring grafts with statistical significance in comparison with males.

**Figure 5:**
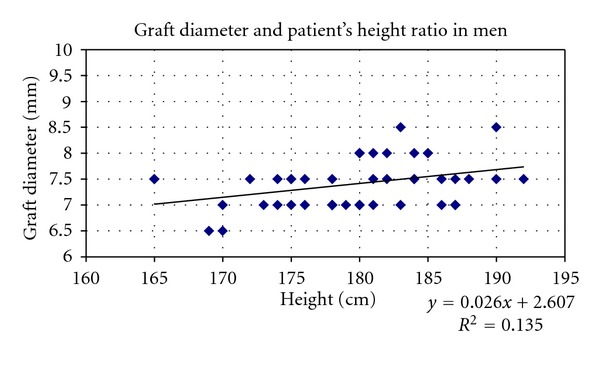
Scatter plots showing relationships between height and hamstring graft size (diameter) in males. Correlation coefficients and *P* values are included. *r* = 0.368239, *r* for *χ* ^2^ = 0.29426, *P* = 0.05.

**Figure 6:**
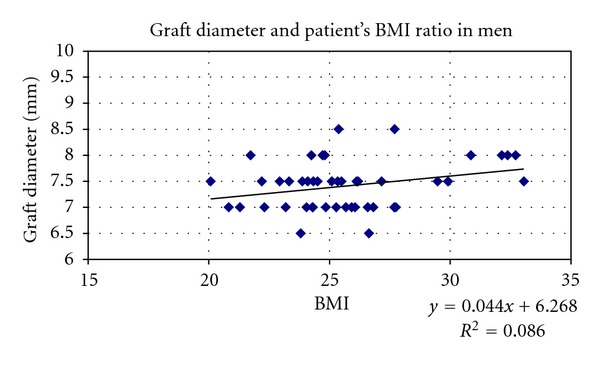
Scatter plots showing relationships between BMI and hamstring graft size (diameter) in males. Correlation coefficients and *P* values are included. *r* = 0.294618, *r* for *χ* ^2^ = 0.25, *P* = 0.01.

Linear regression analyses separated by gender, considering height and BMI had showed existing statistically important correlation with graft size (diameter) only in males (Figures 5 and 6). 

**Figure 7:**
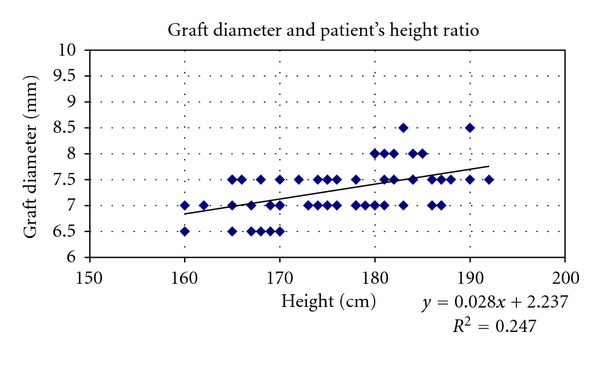
Scatter plots showing relationships between hamstring graft size and patient's height in the whole sample. Correlation coefficients and *P* values are included. *r* = 0.497896, *r* for *χ* ^2^ = 0.32773, *P* = 0.01.

**Figure 8:**
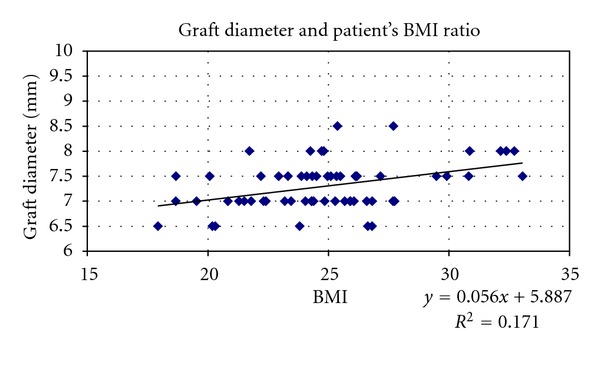
Scatter plots showing relationships between hamstring graft size and patient's BMI in the whole sample. Correlation coefficients and *P* values are included. *r* = 0.413521, *r* for *χ* ^2^ = 0.32773, *P* = 0.01.

Considering the whole sample, hamstring graft size was correlated to patient's height and BMI (Figures 7 and 8).

**Figure 9:**
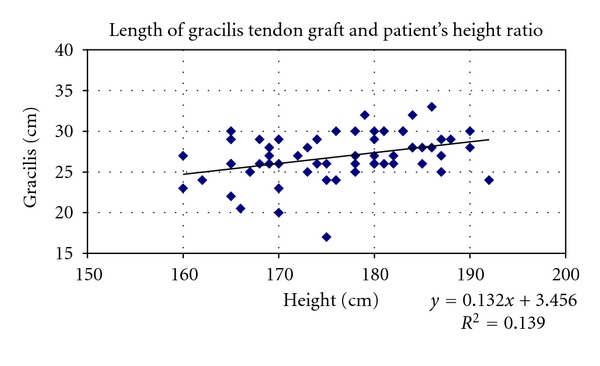
Scatter plots showing relationship between height of patients and length of G tendon graft in the whole sample. Correlation coefficients and *P* values are included. *r* = 0.373363094, *r* for *χ* ^2^ = 0.32773, *P* = 0.01.

**Figure 10:**
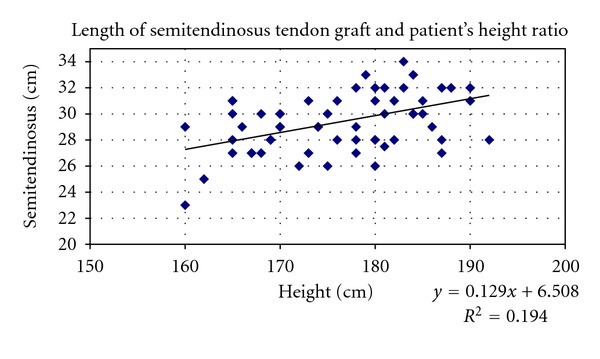
Scatter plots showing relationship between height of patients and length of ST tendon graft in the whole sample. Correlation coefficients and *P* values are included. *r* = 0.441248, *r* for *χ* ^2^ = 0.32773, *P* = 0.01.

Only height of patients was correlated with length of gracilis and semitendinosus graft and not BMI (Figures 9 and 10).

Pearson's correlation tests analysis in the whole sample indicates that hamstring graft size (diameter) was correlated to patient's height, BMI, and also weight. Height and weight of patients was correlated with length of G and ST tendon graft but not BMI (Table 5). According to gender, Pearson's correlation tests analysis considering weight, height, and BMI had shown existing statistically important correlation with graft size (diameter) only in males (Table 6). 

Simple linear regression indicated that patient BMI and height explained approximately 17.1% and 24.7%, respectively, of the variation in quadrupled graft diameter.

Through regression analysis, we constructed the following predictive equations for quadrupled graft diameter:

- diameter = 5.887 + 0.056 (BMI) (*r* = 0.33; *R* ^2^ = 0.171; *P* = 0.01),
- diameter = 2.237 + 0.028 (height in cm) (*r* = 0.33; *R* ^2^ = 0.247; *P* = 0.01).

These equations indicate that patients with BMI less than 19.875 and less than 170 cm tall whose weight is less than 57.4 kg are at highest risk for having a hamstring graft less than 7 mm in diameter.

 Also simple linear regression for graft lengths indicated that height explained approximately 13.9% of variance in G tendon length and 19.4% of the variance in ST length. Through regression analysis, we came up with the following predictive equations for G graft length (GL) and ST graft length (SL):

- GL = 3.456 + 0.132 (height in cm) (*r* = 0.33; *R* ^2^ = 0.139; *P* = 0.01),
- SL = 6.508 + 0.129 (height in cm) (*r* = 0.33; *R* ^2^ = 0.194, *P* = 0.01).

These equations indicate that patients with height less than 167 cm are at highest risk for having an inadequate semitendinosus graft tendon less than 28 cm in length.

When we separated these analyses by gender, we found that height and probably BMI only referring to G length were the best predictors for graft diameter in male patients. We found no statistically important predictor for graft diameter in female patients (Table 6).

## 4. Discussion

In our study in one out of five patients (21%) the length of ST tendon, harvested by the common technique, was inadequate in order to be used alone as a four-strand graft. Especially in female patients, the length of ST tendon was less than 28 cm in 43.75%. Moreover, according to our findings, height and weight are considered to be moderate predictors of the adequacy of the semitendinosus tendon length when using alone ST four-strand graft or of the four-strand ST and G graft diameter for ACL single-bundle reconstruction harvested by common technique (without bone plug). The most reliable predictor seems to be patient's height in males. In female patients, there is no such statistically important predictor. 

The use of ST and G grafts seems to have good results in many studies [18–21], while other studies report similar results by the use of ST only tendon as a quadrupled graft in reconstruction of ACL [22–24]. Gobbi et al. recommended using only one tendon whenever possible because the ST alone seem to have an advantage over the ST-G construct with regard to internal rotation weakness following harvest of two tendons, although there is not much clinical difference in both techniques [12]. In order to assure the optimal 7 cm quadrupled graft construct (2 cm in the femoral tunnel, 3 cm intra articular, and 2 cm in the tibial tunnel), it is essential to obtain a minimum tendon length of 28 cm (ranged from 28 to 30 cm) [11]. Increased research of double-bundle reconstruction and development of new operative techniques necessitate preoperative planning of size parameters for ideal graft choice [25]. Furthermore, a new technique of ACL reconstruction with double-bundle, single tendon (ST) seems to offer the possibility of reconstructing both the AM and PL bundles without disrupting the function of hamstring muscles. This is achieved due to preservation of gracilis tendon, which offers stability in deep flexion and internal rotation strength and protects from further ACL injuries [12]. But even in this case, the minimum graft length needed is 28 cm (2 cm in the femoral tunnel, 3 cm intra-articular, and 2 cm in the tibial tunnel) [12]. Additionally, it has been demonstrated that the average diameter of the normal ACL is 11 mm; therefore, a graft of minimum thickness of 7 mm is recommended [15–17]. The thicker the graft is the stronger and stiffer the graft will be. The biomechanical properties of the graft are certainly affected by its diameter. 

According to Vernon et al., the use of ST tendon alone is adequate in almost all cases [26] and the rate of insufficiency for a quadrupled reconstruction is only one in 300 cases and is almost always the result of improper graft harvest [27]. In contrast to our results regarding the adequacy of semitendinosus tendon as a four-strand graft for ACL reconstruction, the ST graft length was inadequate (i.e., shorter than 28 cm) in 21% of all our patients and in 18% it was marginally adequate (28 cm) and only in 61% of our patients semitendinosus tendon graft length was longer than 28 cm. This is a high percent of possible cases in which ST four-strand tendon graft could be inadequate for ACL reconstruction and additional G tendon graft would be needed and comes in contrast to claims of other authors who support and recommend to use of only one tendon whenever possible [7]. Referring to female patients, these rates are more impressive, while in 43.75% of all cases the ST graft length was inadequate, and in 18.75% it was marginally adequate and only in 37.5% of our female patients ST tendon graft length was longer than 28 cm (Table 3). Additionally simple linear regression for graft lengths indicated that patients with height less than 167 cm are at highest risk for having an inadequate ST graft tendon less than 28 cm in length.

Referring to graft diameter and according to our results, the majority of patients (86.7%) had an adequate quadrupled graft diameter (7 to 8 mm), while 10% of patient's grafts were inadequate (less than 7 mm). Referring to female patients, this percent becomes 25%. Only 2 patients (3.3%) had graft diameter greater than 8 mm (Table 4). Pinheiro et al. report that males with height equal to or greater than 1.80 m achieved a higher percentage of 9 mm grafts and larger average of graft diameter in comparison to the other patients with a height less than 1.80 m males or females or both [28]. In our sample, the two men with graft diameter greater than 8 mm had height greater than 1.80 m (1.83 m and 1.90 m). The hypothesis of Pinheiro et al. [28] is confirmed in our cases, but we cannot reach to safe conclusion because of the small number of our cases.

Hamstring graft size according to our study could be predicted by evaluating preoperatively some simple anthropometric parameters. According to our results ST, and G graft diameter was most strongly correlated to patient's weight (moderate correlation, *r* = 0.567), then to height (moderate correlation, *r* = 0.498) and finally to BMI (fair correlation, *r* = 0.414). Treme et al. in a study of 50 consecutive patients observed a positive effect of the BMI on graft diameter [29] in contrast to Tuman et al. and Pinheiro et al. who claim that BMI does not influence graft diameter [28, 30]. Referring to patient's weight in the study of Pinheiro et al. had less influence in graft diameter, contrary to us and to Treme et al. who found the strongest correlation with weight [28, 29]. Finally Schwatzberg et al. found moderate correlation between weight and graft diameter in a study of 119 consecutive patients [31]. In another series of 536 patients, height was found to be a strong predictor of quadrupled hamstring graft diameter in 234 male patients [32].

The lengths of the hamstring graft can also be predicted by preoperative anthropometric measurements. In our study, the length of G and ST graft was most strongly correlated with height (fair correlation, *r* = 0.441) and then with weight (weak correlation, *r* = 0.369) of patient's, but there was no correlation with BMI. Also in a study of 80 patients, Pinheiro et al. claim that height is the most important variable that influences most the graft length [28]. Treme et al. noted that height and leg length were strongly correlated with the hamstring tendon lengths [30]. Chiang et al. in a study of 100 patients conclude that the patients' height could be used to predict both ST and G tendon lengths in Chinese patients [33]. Tuman et al. after studying 106 patients concluded that height was also the most important variable but mainly in women [30]. Schwatzberg et al. claim weak correlation to patient's height [31]. 

We found no statistically important predictor for graft diameter in female patients. Female patients were significantly lighter and shorter with lower BMIs and had shorter length and smaller diameter hamstring grafts in comparison with males. This result is in accordance with studies of Tuman et al., and Treme et al., who claimed that, mean values of graft diameter as well as weight and height in males were greater than in females [29, 30]. Chiang et al. also in their findings showed that men had significantly longer tendons than women [33]. In our study, simple linear regression for graft diameter indicated that patients with BMI less than 19.875 and less than 170 cm tall whose weight is less than 57.4 kg are at highest risk for having a hamstring graft less than 7 mm in diameter. 

There are some limitations in our study. Firstly, the sample of female patients is not adequate in order to exact secure conclusions. Moreover, we were unable to investigate if smaller size of hamstring graft tendon in women were related to gender or to the smaller average anthropometric measurements [28]. This was due to the fact that our groups according to gender had great differences of anthropometric variances. Secondly, we recognize the fact that our results could be influenced by the size of the sample, which could influence our data and as a consequence our results. For example, the correlation of BMI and the length of the graft could change if we had operated patients with great BMI. However, we believe that this patient group is a representative sample of patients that we operate for ACL deficiency. Thirdly, we did not evaluate the different level of sport activity of our patients and any possible correlation with graft diameter of length. Finally, in some cases the graft diameter was not exactly fitted in a specific 0.5 mm increment. In these cases, the size of the graft was recorded according to the drill size of our tunnels.

The clinical relevance of this study showed that in shorter or female patients, there was a relatively higher risk of obtaining inadequate individual hamstring tendon lengths for double-bundle anterior cruciate ligament reconstruction procedures. Moreover, in our surgical practice, we used to harvest first the G tendon and then the ST tendon. After this study, we have altered our technique. The clinical importance of these findings and our suggestion is that ST tendon graft removal should always be performed before G tendon harvesting, and according to its adequacy of length (>28 cm), the surgeon should decide whether further augmentation of the ACL graft with G tendon would be necessary.

## 5. Conclusions

Hamstring grafts less than 7 mm in diameter and 28 cm in length are not so rare. According to our findings we come to the conclusion that the length of ST tendon, harvested by the common technique, is usually inadequate in order to be used alone as a four-strand graft especially in females. Identification of these patients is still a subject of research. The potential of size prediction of autograft hamstring tendons in ACL reconstruction could contribute to choose the best graft and surgical technique individualized on patient's needs and in accordance with their special characteristics. By that way the possibilities of a good postsurgical result would be multiplied in difficult cases of patients like women, children, and professional athletes or revision of ACL. Also special surgical techniques such as quadrupled ST double-bundle ACL reconstruction and the DBST (double bundle, single-tendon) technique could be used more wisely avoiding unnecessary complications such as scar formation, pain, and operating time and infection risk. Height and weight are considered to be moderate predictors of the adequacy of the semitendinosus tendon length when using alone ST four-strand graft or of the four-strand ST and G graft diameter for ACL single-bundle reconstruction harvested by common technique (without bone plug). The most reliable predictor seems to be patients' height in males. In female patients, there is no such statistically important predictor. 

## Figures and Tables

**Table 1:** Means and standard deviation of demographic data.

	*N*	Age (years)	Height (cm)	Mass (kg)	BMI
Males	45	27.23 ± 6.49	179.73 ± 6.45	68.18 ± 27.23	25.72 ± 3.1
Females	16	24.63 ± 8.63	166.5 ± 3.62	63.13 ± 10.97	22.72 ± 3.59

Total	61	27.02 ± 7.67	176.26 ± 8.26	77.85 ± 14.09	24.93 ± 3.47

**Table 2:** Means and standard deviation of graft data.

	Length of gracilis (G) tendon graft (cm)	Length of semitendinosus (ST) tendon graft (cm)	Diameter of four-strand hamstring autograft (mm) (G-ST)
Males	27.33 ± 2.88	29.94 ± 2.35	7.41 ± 0.47
Females	25.59 ± 2.81	27.81 ± 1.97	7 ± 0.37

Total	26.88 ± 2.94	29.39 ± 2.43	7.30 ± 0.48

**Table 3:** Frequency of adequate ST tendon graft length according to gender.

	Length of semitendinosus (ST) tendon graft <28 cm	Length of semitendinosus (ST) tendon graft 28 cm	Length of semitendinosus (ST) tendon graft >28 cm
	*N* (%)	*N* (%)	*N* (%)
Males	6/45 (13.3%)	8/45 (17.7%)	31/45 (69%)
Females	7/16 (43.75%)	3/16 (18.75%)	6/16 (37.5%)

Total	13/61 (21%)	11/61 (18%)	37/61 (61%)

**Table 4:** Frequency of adequate four-strand hamstring (ST-G) autograft diameter according to gender.

	Diameter of four-strand hamstring (G-ST) autograft <7 mm	Diameter of four-strand hamstring (G-ST) autograft 7-8 mm	Diameter of four-strand hamstring (G-ST) autograft >8 mm
	*N* (%)	*N* (%)	*N* (%)
Males	2/45 (4.4%)	41/45 (91.2%)	2/45 (4.4%)
Females	4/16 (25%)	12/16 (75%)	—

Total	6/61 (10%)	53/61 (86.7%)	2/61 (3.3%)

**Table 5:** Pearson's correlation coefficients of weight, height, and BMI with hamstring tendon graft characteristics in the whole sample.

	Length of gracilis (G) tendon graft	Length of semitendinosus (ST) tendon graft	Diameter of four-strand hamstring (ST-G) autograft
Weight	0.310^∗^	0.369^∗∗^	0.567^∗∗^
Height	0.373^∗∗^	0.441^∗∗^	0.498^∗∗^
BMI	0.165	0.206	0.414^∗∗^

**P < 0.01.

*P < 0.05.

**Table 6:** Pearson's correlation coefficients of weight, height, and BMI with hamstring tendon graft characteristics according to gender.

		Length of gracilis (G) tendon graft	Length of semitendinosus (ST) tendon graft	Diameter of four-strand hamstring (ST-G) autograft
Weight	Male	0.194	0.148	0.470^∗∗^
	Female	0.193	0.266	0.408
Height	Male	0.321^∗^	0.237	0.368^∗^
	Female	0.067	0.378	0.227
BMI	Male	0.025	0.021	0.295^∗^
	Female	0.194	0.210	0.391

**P < 0.01.

*P < 0.05.

## References

[1] Bach BR, Boonos CL (2001). Anterior cruciate ligament reconstruction. *Association of Operating Room Nurses Journal*.

[2] Lephart SM, Kocher MS, Harner CD, Fu FH (1993). Quadriceps strength and functional capacity after anterior cruciate ligament reconstruction. *American Journal of Sports Medicine*.

[3] Freedman KB, D’Amato MJ, Nedeff DD, Kaz A, Bach BR (2003). Arthroscopic anterior cruciate ligament reconstruction: a metaanalysis comparing patellar tendon and hamstring tendon autografts. *American Journal of Sports Medicine*.

[4] Feller JA, Webster KE (2003). A randomized comparison of patellar tendon and hamstring tendon anterior cruciate ligament reconstruction. *American Journal of Sports Medicine*.

[5] Hamner DL, Brown CH, Steiner ME, Hecker AT, Hayes WC (1999). Hamstring tendon grafts for reconstruction of the anterior cruciate ligament: biomechanical evaluation of the use of multiple strands and tensioning techniques. *Journal of Bone and Joint Surgery A*.

[6] Woo SLY, Kanamori A, Zeminski J, Yagi M, Papageorgiou C, Fu FH (2002). The effectiveness of reconstruction of the anterior cruciate ligament with hamstrings and patellar tendon: a cadaveric study comparing anterior tibial and rotational loads. *Journal of Bone and Joint Surgery A*.

[7] Williams RJ, Hyman J, Petrigliano F, Rozental T, Wickiewicz TL (2004). Anterior cruciate ligament reconstruction with a four-strand hamstring tendon autograft. *Journal of Bone and Joint Surgery A*.

[8] Gobbi A, Domzalski M, Pascual J, Zanazzo M (2005). Hamstring anterior cruciate ligament reconstruction: is it necessary to sacrifice the gracilis?. *Arthroscopy*.

[9] Gobbi A, Francisco R Anatomic Double Bundle ACL Reconstruction with the Semitendinosus Tendon.

[10] Makihara Y, Nishino A, Fukubayashi T, Kanamori A (2006). Decrease of knee flexion torque in patients with ACL reconstruction: combined analysis of the architecture and function of the knee flexor muscles. *Knee Surgery, Sports Traumatology, Arthroscopy*.

[11] Gobbi A, Francisco R (2005). Quadruple semitendinosus tendon for anterior cruciate ligament reconstruction. *Techniques in Orthopaedics*.

[12] Gobbi A Double bundle ACL reconstruction Using Only the Semitendinosus.

[13] O’Neill DB (2004). Revision arthroscopically assisted anterior cruciate ligament reconstruction with previously unharvested ipsilateral autografts. *American Journal of Sports Medicine*.

[14] Scott WN, Insall JN, Rockwood CA, Green DP, Bucholz RW (1996). Injuries of the knee. *Rockwood and Green’s Fractures in Adults*.

[15] Feller JA, Siebold R, Webster KE (2005). ACL reconstruction in females: patellar tendon versus hamstring tendon autograft. *Journal of Bone and Joint Surgery B*.

[16] Grood ES, Walz-Hasselfeld KA, Holden JP (1992). The correlation between anterior-posterior translation and cross-sectional area of anterior cruciate ligament reconstructions. *Journal of Orthopaedic Research*.

[17] Hamada M, Shino K, Horibe S, Mitsuoka T, Toritsuka Y, Nakamura N (2005). Changes in cross-sectional area of hamstring anterior cruciate ligament grafts as a function of time following transplantation. *Arthroscopy*.

[18] Marcacci M, Zaffagnini S, Iacono F (2003). Intra- and extra-articular anterior cruciate ligament reconstruction utilizing autogeneous semitendinosus and gracilis tendons: 5-Year clinical results. *Knee Surgery, Sports Traumatology, Arthroscopy*.

[19] Howell SM, Deutsch ML (1999). Comparison of endoscopic and two-incision techniques for reconstructing a torn anterior cruciate ligament using hamstring tendons. *Arthroscopy*.

[20] Howell SM Gold standard-DLSTG graft.

[21] Beynnon BD, Johnson RJ, Fleming BC (2002). Anterior cruciate ligament replacement: comparison of bone-patellar tendon-bone grafts with two-strand hamstring grafts: a prospective, randomized study. *Journal of Bone and Joint Surgery A*.

[22] Gobbi A, Tuy B, Panuncialman I, Mahajan S (2003). Quadrupled bone-semitendinosus anterior cruciate ligament reconstruction: a clinical investigation in a group of athletes. *Arthroscopy*.

[23] Cooley V, Deffner K, Rosenberg T (2001). Quadrupled semitendinosus anterior cruciate ligament reconstruction: 5-Year results in patients without meniscus loss. *Arthroscopy*.

[24] Goradia V, Grana W (2001). A comparison of outcomes at 2 to 6 years after acute and chronic anterior cruciate ligament reconstructions using hamstring tendon grafts. *Arthroscopy*.

[25] Buoncristiani AM, Tjoumakaris FP, Starman JS, Ferretti M, Fu FH (2006). Anatomic double-bundle anterior cruciate ligament reconstruction. *Arthroscopy*.

[26] Vernon C, Kathleen D, Thomas R (2001). Quadrupled semitendinosus anterior cruciate ligament reconstruction: 5-Year results in patients without meniscus loss, arthroscopy. *The Journal of Arthroscopic and Related Surgery*.

[27] Cooley V, Deffner K (2005). Quadrupled semi-T anterior cruciate ligament reconstruction: tips, techniques, and results. *Techniques in Orthopaedics*.

[28] Pinheiro B, Percope A, Teixeira M (2011). Intra-operative four-stranded hamstring tendon graft diameter evaluation. *Knee Surgery, Sports Traumatology, Arthroscopy*.

[29] Treme G, Diduch DR, Billante MJ, Miller MD, Hart JM (2008). Hamstring graft size prediction: a prospective clinical evaluation. *American Journal of Sports Medicine*.

[30] Tuman JM, Diduch DR, Rubino LJ, Baumfeld JA, Nguyen HS, Hart JM (2007). Predictors for hamstring graft diameter in anterior cruciate ligament reconstruction. *American Journal of Sports Medicine*.

[31] Schwartzberg R, Burkhart B, Lariviere C (2008). Prediction of hamstring tendon autograft diameter and length for anterior cruciate ligament reconstruction. *American Journal of Orthopedics*.

[32] Ma CB, Keifa E, Dunn W, Fu FH, Harner CD (2010). Can pre-operative measures predict quadruple hamstring graft diameter?. *Knee*.

[33] Chiang ER, Ma HL, Wang ST, Hung SC, Liu CL, Chen TH (2012). Hamstring graft sizes differ between Chinese and Caucasians. *Knee Surgery, Sports Traumatology, Arthroscopy*.

