# Erratum to “Adequacy of Semitendinosus Tendon Alone for Anterior Cruciate Ligament Reconstruction Graft and Prediction of Hamstring Graft Size by Evaluating Simple Anthropometric Parameters”

**DOI:** 10.1155/2013/925480

**Published:** 2013-04-10

**Authors:** S. G. Papastergiou, G. A. Konstantinidis, K. Natsis, E. Papathanasiou, N. Koukoulias, A. G. Papadopoulos

**Affiliations:** ^1^Orthopaedic Department of “Saint Paul” General Hospital of Thessaloniki Greece, Ethnikis Antistaseos 161, 55134 Thessaloniki, Greece; ^2^Department of Anatomy, Medical School, Aristotle University of Thessaloniki, University Campus, P.O. Box 300, 54624 Thessaloniki, Greece

The authors' names were incorrectly listed as Papastergiou G. Stergios, Konstantinidis A. Georgios, Natsis Konstantinos, Papathanasiou Efthymia, Koukoulias Nikolaos, and Papadopoulos G. Alexandros; this error is corrected here.

